# Selected issues in the anatomy and physiology of the avian organ of vision and eye disorders in farmed poultry

**DOI:** 10.2478/jvetres-2025-0034

**Published:** 2025-06-11

**Authors:** Andrzej Koncicki, Marta Pietruszyńska, Martyna Mieszczak, Joanna Stępniewska, Bartłomiej Tykałowski, Tomasz Stenzel

**Affiliations:** 1 Department of Poultry Diseases, Faculty of Veterinary Medicine, University of Warmia and Mazury in Olsztyn, 10-719 Olsztyn, Poland; 2 Department of Clinical Pharmacology, Medical University of Bialystok, 15-274 Białystok, Poland; 3 Clinical Department of Ophthalmology with Pediatric Ophthalmology Subdivision, University Clinical Hospital in Poznan, 60-355 Poznań, Poland

**Keywords:** avian eye, eye anatomy, eye physiology, pathological conditions, farmed poultry

## Abstract

The aim of this article was to review current knowledge of the anatomy and physiology of the avian eye and ocular diseases in poultry. The avian visual organ consists of the eye and extraocular organs and, unlike the mammalian eye, is characterised by many structural and functional adaptations that enhance vision. Avian eyes do not have the same functions as mammalian eyes. Bird eyes have high sensory sensitivity and are capable of constellation recognition for star orientation and navigation; they may be regarded as the finest ocular organs in the animal kingdom. It is generally accepted that the considerable differences in the morphology and function of avian eyes are largely due to adaptations to specific activities and environmental conditions. Eye diseases are rarely diagnosed in poultry because, unlike livestock and pets, detailed ophthalmological examinations are not carried out on farmed birds. Nevertheless, poultry are frequently affected by ocular pathologies, including developmental abnormalities, infectious and non-infectious diseases, degenerative processes, tumours, injuries and pathological conditions of unknown aetiology. In poultry, ocular disease is often associated with respiratory and systemic disease. Ocular pathology in farmed birds has a complex aetiology and its incidence can be reduced by controlling the incubation parameters of hatching eggs, avoiding feeding errors in breeder flocks and rearing poultry under conditions that optimise welfare and comply with biosecurity standards.

## Introduction

Birds’ eyes are the finest ocular organs in the animal kingdom, being capable of constellation recognition for stellar orientation and navigation. In birds, the eye is the most important sensory organ, and other senses can compensate for vision impairments only to a limited degree. This applies particularly to wild birds, whose flying and foraging capabilities depend largely on the sense of sight. Even partial or minor vision impairments can have far-reaching consequences in wild birds (57). In contrast to wild birds’ survival, that of farmed poultry or companion birds kept in cages or aviaries is not undermined by eye disorders or diseases. Neither are vision impairments health conditions that critically affect farmed-bird performance or farm efficiency. In addition, farmed poultry have a relatively short lifespan. For these reasons, eye diseases and diagnostic methods have been more extensively studied in companion birds (60) than in farmed poultry. The aim of this paper was to review the current knowledge of the anatomy and physiology of the avian eye and eye diseases in poultry, particularly chickens, turkeys and waterfowl raised on farms.

### Anatomy and physiology of the avian organ of vision

The avian organ of vision consists of the eye and extraocular organs, and in contrast to the mammalian eye, it is characterised by many structural and functional adaptations that enhance vision for high sensitivity. Avian eyes do not have the same functions as mammalian eyes. It is generally believed that the considerable differences in the morphology and function of avian eyes can be largely attributed to adaptations to a specific activity and environmental conditions (54). The size and weight of the avian eye testify to the importance of this sensory organ in birds. The size of the eye is correlated with the length of the eyeball, which ranges from around 8 mm in the kiwi (*Apteryx* spp.), a bird species with a relatively low head weight, to 50 mm in the ostrich (*Struthio camelus*), which has the largest eyes of all present vertebrates. The eyes account for approximately 7% of total head weight in adult chickens, 12% in one-day-old chicks, up to 32% in some owls, but only around 1% in humans (5).

The sclera is the fibrous outer coat of the eye that consists of scleral cartilage, scleral ossicles and the cornea. Ten to eighteen scleral ossicles form a ring of overlapping plates near the corneal limbus. The avian cornea is thinner than the mammalian cornea. The cornea, the refractive element of the eye, is composed of tightly woven collagen fibres that are responsible for the mechanical resistance and transparency of the front layer of the eye. The cornea does not have blood vessels, and nutrients are supplied by diffusion from the aqueous humour and tear film. The anterior surface of the cornea is covered by several layers of stratified squamous epithelium resting on the basement membrane (Bowman’s membrane). The innermost surface of the cornea is covered by a monolayer of simple squamous epithelial cells known as the corneal endothelium, which is separated from the corneal stroma by a thick basement membrane (Descemet’s membrane). The cornea is convex in raptors and flattened in aquatic birds (5, 57, 60).

The shape of the avian eye is a species-specific trait, and it may be flat (in diurnal passerines and aquatic birds), globose (in diurnal birds of prey) or tubular (in nocturnal raptors such as owls) (57, 60). The shape of the eye is determined by cartilage embedded in the posterior part of the sclera and scleral ossicles that are positioned caudally to the corneal limbus and form a ring of osseous plates. The scleral ring protects the eye, in particular in birds with flat orbits (such as owls), and is responsible for the corneal curvature (60).

The uvea is the middle vascular layer of the eye that consists of the iris, ciliary body and choroid. The iris contains lipochrome pigments, the colour of which differs by age and sex in various bird species (60). Unlike some mammals, almost all birds do not have a *tapetum lucidum*, a layer of shiny tissue that reflects light. A *tapetum lucidum* composed of iridocytes is only found in birds of the *Columbiformes* order. In birds, the iris musculature is composed mainly of striated muscles (47). The muscles of the ciliary body consist of the anterior ciliary muscle which increases the corneal curvature and the posterior ciliary muscle which increases the curvature of the eye lens. Most birds also have a sphincter muscle and a set of dilator muscles that control pupil size. The choroid layer, which lines the entire back of the eyeball, features large blood vessels and a dense network of capillaries (47).

The anatomy of the avian eye is unique for several reasons. The avian retina resembles other vertebrate retina in terms of neural organisation, but it is thicker and devoid of vasculature. The retina is nourished mainly by the choroid. The retinal pigment epithelium contains more pigment particles in diurnal birds and fewer pigment particles in nocturnal birds (such as owls). The avian retina is composed of rods and cones, and the visual acuity of birds is determined by the density of functional photoreceptors that contain photopigments, including rhodopsin (found in rods) and opsin (found in cones). Rod cells are responsible for visual perception under low-light conditions (scotopic vision), whereas cone cells are associated with colour vision and perception of fine detail. Cone cells contain oil droplets with spectral filtering properties, which is why birds can discriminate colours more effectively than humans (8, 29). Clusters containing different numbers of cones in different species are located in foveal pits that allow light to directly stimulate the cones (60). In diurnal birds, the *fovea centralis* in the central part of the retina is responsible for acute monocular vision, whereas the *foveae* in the temporal retina are responsible for binocular vision and accommodation for near objects. The avian *fovea* contains up to 400,000 cones per square millimetre, and cone density is twice as high as in humans. In the Eurasian goshawk (*Astur gentilis*), cone density is even higher, reaching one million per square millimetre. Because of their high density of photoreceptors, birds can perceive far more details in the spatial dimension than humans (8, 29, 60).

Ultraviolet (UV) light perception is a specific adaptive trait in wild birds. Most diurnal birds are capable of detecting light in the UV spectrum of 320 to 680 nm (400–600 nm in humans), which promotes intraspecies and interspecies communication. Ultraviolet light is reflected from the plumage, and birds that appear monomorphic to the human eye rely on UV light to distinguish between the sexes. Ultraviolet light perception also enables birds to find food and identify fruit ripeness based on varying UV-reflecting properties of fruit wax layers (8, 57). The high spatial frequency of the avian retina is yet another adaptive trait of wild birds. Birds can process up to 160 frames per second, which is 2–4 times more than mammals (and over 10 times more than humans’ 10–15 frames per second) and enables birds to detect small animals (a potential source of food) from flight altitudes. In addition, wild birds can also spot very slow motion, which plays an important role in detecting insects as a food source and evading ambush predators. Chickens have a sight range of up to 50 m, geese one of up to 120 m, and ducks up to 80 m. Some species of wild birds have a nearly 360-degree field of vision, far superior to the human visual field extending from 85 to 100 degrees temporally, and are able to see the entire space around the head (full-circle vision). Visual accommodation ranges from around 40 diopters in parrots and raptors to nearly 80 diopters in diving birds, which compensates for the loss of the cornea’s refractive power underwater.

Birds detect light not only through photoreceptor cells in the retina, but also through photosensitive cells in the pineal gland. In most birds, the pineal gland is both a receptor and a secretory organ (46). Pinopsin is a light-sensitive pigment that is characteristic of the avian pineal gland and is found in the apical part of rudimentary-receptor pinealocytes and secretory pinealocytes. The avian pineal gland expresses the gene encoding melanopsin, a type of photopigment that is found in retinal ganglion cells in mammals and plays an important role in regulating circadian rhythms (30). As a result, the avian pineal gland can convert light signals into chemical signals by synthesising and secreting melatonin into the bloodstream. Melatonin is released in response to darkness, and it synchronises the endogenous circadian rhythm with external cues (30, 42).

The avian lens and the mammalian lens have a similar anatomy – both are biconvex in shape and have a similar refractive index. However, the avian lens tends to be more convex, which increases refractive power. Aquatic birds have more flexible lenses that enhance vision acuity in both air and water. Flexible lenses promote optical accommodation for good visual performance at various distances and in media with different refractive indices (air and water). The avian lens is soft with a harder outer layer that is responsible for rapid accommodation. In the mammalian eye, the range of accommodation is controlled by changing the shape of the lens, whereas birds have an additional set of muscles that can change the shape of the cornea. The lens divides the eye into anterior and posterior chambers. The anterior chamber between the lens and the cornea is filled with the aqueous humour. Similarly to the mammalian eye, the posterior chamber contains the vitreous humour, a gelatinous substance composed mainly of water (99%), collagen, and hyaluronic acid. However, the *pecten oculi* is a unique feature of the avian eye. The vitreous humour absorbs some shocks and allows the passage of light to the retina. The optic nerve is well developed and connects to the ventroposterior part of the eyeball (5, 57, 60). The *pecten oculi* is a pigmented comb-like structure that is unique to the avian eye. It consists of connective tissue, neuroglial cells and parallel blood vessels, and projects into the vitreous humour at the point where the optic nerve enters the eyeball (6). It is believed that the *pecten oculi* nourishes the avascular retina *via* the vitreous humour and regulates intraocular pressure and pH (7, 20).

In birds, the upper and lower eyelids are thin, sometimes transparent, and bristled. Birds also have a translucent third eyelid known as the nictitating membrane. The membrane is a fold of bulbar conjunctiva in the inner corner of the eye. The avian eye also features thin and weakly developed rectus and oblique muscles, the lacrimal apparatus with the gland of the nictitating membrane, and two muscles that control the third eyelid (quadratus and pyramidalis muscles). Birds do not have muscles that retract the eyeball into the skull or tarsal glands (Meibomian glands). They compensate for reduced eyeball mobility by moving their heads. The Harderian gland that facilitates the movement of the nictitating membrane is very well developed in birds. The lacrimal gland is located in the ventrotemporal part of the orbit and is covered by the bulbar conjunctiva. The nasal (salt) gland is best developed in marine birds, where it excretes hypertonic sodium solution, and it is positioned in the orbit, dorsomedially to the eyeball. The anatomy of the conjunctiva is similar in birds and mammals. The conjunctiva-associated lymphoid tissue, the Harderian gland, lacrimal glands, and the nasolacrimal duct play a role in avian adaptive immunity (5, 57, 60). The avian orbit is usually large, but it is not completely encompassed by bone. Nasal and paranasal sinuses are located in close proximity to the orbits, which is why infections of the upper respiratory tract often spread to the orbits and the eyes (60).

### Pathological conditions of the organ of vision of farmed poultry

Unlike in mammals, ophthalmological disorders are rarely diagnosed in poultry and are usually detected during clinical examinations of flocks. Detailed ophthalmological examinations are performed on ornamental birds, but not on farmed birds. However, poultry are often affected by pathologies of the visual organs, including developmental anomalies, infectious and non-infectious diseases, degenerative processes, tumours, injuries and pathological conditions of unknown aetiology. In poultry, eye disorders often accompany respiratory and systemic diseases (55, 57).

Congenital eye anomalies include retinal dysplasia and abnormal retinal development. These anomalies have been described in both laying hens and broiler chickens (55, 56). Congenital eye anomalies can affect chicks in the first days of life, and their symptoms intensify with age. Chicks with retinal dysplasia tend to be smaller than their healthy counterparts and manifest the clinical signs of being unable to find feed and water and displaying disoriented locomotion. Anatomopathological examinations of chicks with these disorders did not reveal any pathological changes in the eyes. However, microscopic examinations revealed degeneration of photoreceptor cells (cones and rods) in early stages of the disease, followed by rosette formation of the retina, disorganisation of retinal layers, proliferation of retinal pigment epithelial cells and inflammation of the choroid in successive stages. Retinal detachment, cataracts, fibrosis and metaplastic changes in the *pecten oculi* were observed in several-week-old chicks (56, 57).

Developmental anomalies such as ocular albinism and the associated refractive errors (49), ectatic disorders of the cornea (including keratoconus) and sclera, glaucoma (57), microphthalmia (a developmental disorder in which one or both eyes have anatomic malformations) (24), cyclopia, three eyes, anophthalmia (absence of one or both eyes), blindness, buphthalmos, retinopathy, macular edema and optic nerve hypoplasia have been reported in chicks, poults and commercial poultry flocks (4, 32, 55, 57). These anomalies can be genetically conditioned, but in most cases, they are caused by suboptimal incubation conditions, including inappropriate temperature and humidity, or failure of egg-turning devices in incubators (27, 55, 57).

Ocular lymphoma is the most extensively studied eye neoplasm in birds, and it is most prevalent in chickens with Marek’s disease (26). Other eye neoplasms are rarely noted in farmed poultry. Several cases of lymphocytic neoplasms affecting different parts of the eyeball and extraocular tissues, rhabdomyosarcoma in the anterior uvea or the orbit, retinoblastoma, uveal melanoma and neurofibromatosis of the vitreous body have also been described in the literature (57).

Many eye disorders are caused by non-infectious factors, including vitamin deficiencies and inadequate rearing conditions that compromise the welfare of poultry. Significant vitamin A deficiencies or toxic levels of vitamin A in feed cause characteristic pathological changes in the eye. Beta-carotene present in feed is converted to retinol, and retinoic acid induces the differentiation of epithelial cells into goblet cells characteristic of soft and moist epithelial tissue. In vitamin A deficiency, basal cells are differentiated into squamous epithelial cells (squamous metaplasia), which may become keratinised and form a hard and dry epithelium that is typically encountered on skin. Therefore, vitamin A deficiency affects the conjunctival epithelium. In addition, retinal, the aldehyde form of retinol, binds covalently to opsins to form the light-sensitive pigment rhodopsin in retinal rod cells and plays an important role in perception of light, in particular low-intensity light. The disorders caused by vitamin A deficiency can also lead to nyctalopia (night blindness). Low levels of vitamin A can also cause periorbital oedema. During prolonged vitamin A deficiency, oily and milky white secretions accumulate in the eye, and they can cause the eyelids to stick (33). Excessive vitamin A intake can also be harmful, contributing to conjunctivitis, sticky eyelids, and lesions in the corners of the oral cavity (35).

Vitamin E deficiency can lead to the death of embryos on the fourth day of incubation. Corneal opacity, vascular abnormalities and intraocular haemorrhaging have been reported in these embryos. These changes can affect both eyes and lead to blindness in hatched poults. In young turkeys, riboflavin deficiency can contribute to the formation of scabs in the corners of the oral cavity and on the eyelids (pellagra). A deficiency of pantothenic acid can also promote the formation of scabs in the corners of the oral cavity and on the eyelids, and excessive secretions that cause eye stickiness, which decreases vision acuity (33).

Ammonia levels should not be higher than 26 ppm on poultry farms, but they can exceed 100 ppm in poorly ventilated facilities with straw litter. High ammonia concentrations (50–75 ppm) induce pathological changes in the upper respiratory tract and the eyes (21). Ammonium hydroxide, a strong irritant, is produced when ammonia comes into contact with moist conjunctiva, which leads to keratitis and conjunctivitis. Prolonged exposure to ammonia levels higher than 100 ppm can cause epithelial necrosis, corneal ulceration and oedema, photophobia and blindness (33). Uveitis with infiltration of heterophils and lymphocytes in the iris and the ciliary body has been reported in chronic cases. These changes can be exacerbated by secondary bacterial infections (57).

Enzootic eyelid dermatitis and conjunctivitis manifested by secretions from the conjunctival sac, eyelid swelling and ulceration, conjunctival necrosis, lens clouding and blindness, have been observed in female turkeys in breeder flocks. These changes can affect one or both eyes, and they are most prevalent in cool seasons of the year. The aetiology of these disorders has not been fully elucidated, but it is believed that genetic predisposition to allergies (allergic conjunctivitis associated with the expression of the Toll-like 2 receptor), reproductive hormones, vitamin A deficiency, pathogens (*Staphylococcus hyicus*), poor housing conditions such as low air qualityand high dust levels, and the stressing of females by their handling during insemination can increase the associated risks (15, 24).

Unsurprisingly, the lighting conditions in housing for birds may also cause poultry eye disorders. Turkey blindness syndrome, manifested by chorioretinitis and retinitis, is caused by continuous exposure to artificial light over a period of six weeks (19). Broilers reared in dark-out housing for 10 weeks and exposed to low-intensity light (3.2 lux) for six hours per day (18) manifested ophthalmopathy at 22 weeks, and histopathological sections of the eyes revealed retinal degeneration and detachment.

Cataracts of varied aetiology have been described in many bird species. In birds, they may be caused by viral infections such as avian encephalomyelitis, Newcastle disease and Marek’s disease; aspergillosis, toxoplasmosis, nutritional deficiencies (vitamin E deficiency), congenital factors, aging or other disorders of unknown aetiology (26, 28, 32, 57). The observed changes differ in severity, ranging from mild lens fibre degeneration to epithelial hyperplasia with nuclear liquefaction in advanced stages of disease. Cataracts, retinal abnormalities and optic nerve hypoplasia have been described in one-day-old poults. Microscopic examinations revealed fibre degeneration throughout the lens and vacuolisation of epithelial cells with nuclear liquefaction. Visible thinning of retinal ganglion cell walls and optic nerve fibres was observed. These changes were probably caused by congenital ocular syndrome (4).

Infectious diseases, in particular respiratory infections, often lead to eye conditions in poultry. Many viral infections manifest with conjunctivitis, foamy eye discharge from the conjunctival sac and infraorbital sinusitis. These symptoms have been observed in infectious laryngotracheitis in chickens, Newcastle disease, avian influenza, infectious bronchitis, metapneumovirus infections (turkey rhinotracheitis and swollen head syndrome in chickens), circovirus infections and duck viral enteritis (11, 12, 13, 14, 31, 41, 50, 52, 53).

In chickens with infectious laryngotracheitis, conjunctivitis can manifest as a mild lymphocytic inflammation with chemosis, often haemorrhagic lesions with numerous scyntial cells and eosinophilic type B intranuclear inclusion bodies, as well as epithelial necrosis (3, 52). Such advanced inflammatory changes in the conjunctiva have been reported in birds with velogenic Newcastle disease and highly pathogenic avian influenza (11, 14). Conjunctivitis is also one of the main symptoms of infectious bronchitis in chickens. Viral conjunctivitis may also be accompanied by lymphoplasmacytic iridocyclitis (31). Infections with the fowl pox virus can lead to the hypertrophy and proliferation of conjunctival epithelial cells, many of which contain eosinophilic intracytoplasmic inclusion bodies. In some cases, fowl pox can also induce pathogenic changes in the cornea (38).

In the course of Marek’s disease in chickens, macroscopic changes may be observed in the visual organ, ranging from irregular pupil shape due to iris inflammation or discolouration due to loss of pigmentation (“grey eye”) as a consequence of tumour-like lymphocyte infiltration. In advanced stages of the disease, neoplastic changes may spread to the sclera. In chickens, Marek’s disease can lead to choroiditis, corneal oedema, keratitis with eosinophilic intranuclear inclusion bodies in mononuclear cells of the cornea and retinal ganglion cells, necrotising retinitis and cataracts (26). The immune response elicited by the pp38 antigen in Marek’s disease leads to infiltration with CD4^+^ and CD8^+^ T cells, macrophages, heterophils, plasma cells and granulocytes (44).

Many viral diseases can be complicated by secondary bacterial infections, leading to conjunctivitis, keratitis, iritis, scleritis and panophthalmitis (57). In poultry, eye disorders can also be caused by primary bacterial infections, in particular sepsis. Conjunctivitis and swelling of the infraorbital sinuses have been noted in both free-living songbirds and farmed birds infected with *Mycoplasma gallisepticum* (22, 40). Keratoconjunctivitis, increased lacrimation, foamy discharge from the conjunctival sac, epithelial hyperplasia, lymphocytic infiltration and eyelid swelling have been reported in caged layer hens infected with this bacterium (40). Keratoconjunctivitis, eyelid swelling and purulent discharge from the conjunctival sac can also be observed in birds infected with *Chlamydia psittaci*, in particular turkeys and ducks (25). In turkeys, *Salmonella arizonae* infections typically lead to endophthalmitis with fibrinopurulent exudate, superficial punctate keratitis, choroiditis, retinal damage and blindness (58). Infections with *E. coli* can cause keratitis, uveitis and hyphaema in poultry. Septicaemia caused by *E. coli* can lead to panophthalmitis, a purulent inflammation of the eyeball (39). Conjunctivitis, infraorbital sinusitis and swelling of soft head tissues have been reported in turkeys and ducks with pasteurellosis caused by the *Pasteurella multocida* pathogen (43). Conjunctivitis with foamy discharge from the conjunctival sac and swelling of the infrontal sinuses have also been noted in turkeys infected with *Bordetella avium, Ornithobacterium rhinotracheale, Erysipelothrix rhusiopathiae* and *Klebsiella* spp., and in ducks and turkeys infected with *Riemerella anatipestifer* (16, 37, 45, 51, 59). Acute keratoconjunctivitis and retinitis with retinal tears, panophthalmitis, infraorbital sinusitis and swelling of soft head tissues have also been reported in turkeys infected with *Pseudomonas aeruginaosa* (34).

Fungal keratitis caused by *Aspergillus fumigatus* has been described in chickens and turkeys (2). The disease can affect one or both eyes, and it can lead to keratoconjunctivitis with mucous discharge from the conjunctival sac and eyelid swelling. In these cases, the eye infection is caused by inhalation of fungal spores from contaminated litter (23). In turn, in allergic aspergillosis of the respiratory tract, the eye can become infected by fungal spores that are released into the bloodstream. The infection can spread to the internal structures of the eye, and it can affect the retina, iris or even the *pecten oculi* (48). Heterophil and macrophage infiltration was noted in the retina and *pecten oculi*, and granuloma formation was observed in the *pecten oculi*. Fungal fragments were detected in the chambers of the eye and in the retina (48). Infections with *Candida albicans* caused corneal oedema and heterophilic and lymphocytic infiltration in the uvea, cornea and iris (17). In turn, infections with *Microsporum gallinae* led to eyelid swelling, hyperkeratosis, epidermal degeneration and necrosis (10).

Conjunctivitis and inflammations of other structures of the eye can be caused by roundworms (*Oxyspirura mansoni*), flukes (*Philophthalmus gralli*) and protozoans (*Cryptosporidium baileyi* and *Toxoplasma gondii*) (1, 9, 35, 57). Ocular toxoplasmosis caused by *Toxoplasma gondii* cysts and free tachyzoites can lead to optic atrophy and pathological changes in the eye. Cysts can induce inflammatory and degenerative changes in the macula, iris, vitreous humour, choroid, retina and *pecten oculi*, and can cause cataracts (9, 57).

## Conclusion

Pathological eye conditions in farmed birds have a complex aetiology, and their incidence can be reduced by controlling hatching egg incubation parameters, avoiding feeding errors in breeder flocks, and rearing poultry under conditions that optimise welfare and are consistent with biosecurity standards. Exercise of such care should minimise the risk of eye disorders in developing embryos and hatchlings.
